# Digestibility and microbiota modulation by nuts and sunflower seeds in cystic fibrosis: an in vitro approach

**DOI:** 10.1007/s00394-026-04006-7

**Published:** 2026-06-06

**Authors:** Jazmín Viteri-Echeverría, Joaquim Calvo-Lerma, Ana Heredia, Ana Andrés, Andrea Asensio-Grau

**Affiliations:** 1 Food UPV, Universitat Politècnica de València, Camino de Vera S/N, 46022 València, Spain; 2 Present Address: ALISOST Group, Faculty of Pharmacy and Food Sciences, University of Valencia, Avinguda Vicent Andrés Estellés, 46100 València, Spain

**Keywords:** Dietary recommendations, Almond, peanut, hazelnut, short-chain fatty acids, Children, Pediatrics, Lipids, Protein

## Abstract

**Purpose:**

This study evaluated the lipid and protein digestibility and the prebiotic potential of almonds, hazelnuts, peanuts, and sunflower seeds through static in vitro gastrointestinal digestion and colonic fermentation, comparing healthy and cystic fibrosis (CF) conditions.

**Methods:**

The CF model included reduced pH, low bile salt concentration, and pancreatic enzyme replacement therapy. Digestibility was assessed via matrix degradation, proteolysis, and lipolysis, while microbiota composition and metabolic activity were analysed using faecal inoculum from paediatric CF donors.

**Results:**

Sunflower seeds and peanuts showed the highest lipolysis under CF conditions (46%), with sunflower seeds also achieving the highest proteolysis (457 mg tyrosine/g food). Beta-diversity analyses revealed significant differences in microbiota composition after simulated colonic fermentation between the four food samples, with sunflower seeds uniquely reducing *Acidaminococcus* and increasing the production of linear-chain short-chain fatty acids and lactate. In contrast, almonds exhibited notably low proteolysis, potentially due to antinutritional factors.

**Conclusion:**

These findings highlight the differential digestibility and microbiota modulation of nuts and seeds under CF-specific conditions, with sunflower seeds emerging as a promising dietary component for children with CF.

**Supplementary Information:**

The online version contains supplementary material available at 10.1007/s00394-026-04006-7.

## Introduction

Nuts and seeds could contribute to achieving the nutritional goals related to lipids, protein and fibre because of they contain in high amounts these nutrients in different proportions and compositions [1, 2]. In the case of children with cystic fibrosis (CF), the daily intake of nuts and seeds could contribute to achieving their nutritional needs towards improving nutritional status [3].

In normal conditions, dietary triglycerides are hydrolysed mainly by pancreatic lipase, resulting in 2-monoglycerides and free fatty acids, which are absorbable species. In CF, however, exocrine pancreatic insufficiency impairs lipolysis by lack of pancreatin secretion into the duodenum along with reduced bile salts concentration and intestinal pH [4]. To palliate this affection, patients need pancreatic enzyme replacement therapy, which consists of oral supplementation with encapsulated pancreatin in every meal [3]. Complementarily, the high-fat, high-energy diet has been a mainstream recommendation in the nutritional treatment of this pathology, to compensate for fat and energy loss derived from maldigestion [5]. However, it has been repeatedly contrasted that children with CF cover the high lipid intake by consuming low-health profile foods with a high saturated fatty acids content and low fibre content [6]. In the long run, having followed this type of diet leads to the onset of complications, including the promotion of type-2 diabetes and even overweight and obesity [3]. In turn, the colonic microbiota of children with CF is known to present with dysbiosis, which is defined as the unbalanced proportion between beneficial and pathogenic bacteria and the low production of short-chain fatty acids (SCFA). Among the several factors contributing to dysbiosis (recurrent antibiotic use, and altered characteristics), lipid and fibre intake have been proposed as determinant factors [7].

Following the trend toward healthier food consumption, among the alternatives to ultra-processed snacks, nuts and seeds stand out for their nutritional value and their demand is on the rise. Therefore, as concluded in previous work [8], nuts and seeds could entail a dietary strategy to both meet high-fat-diet requirements with a healthy profile and improve dysbiosis of colonic microbiota composition because of the presence of prebiotic compounds such as fermentable fibre. The potential prebiotic impact of nuts and seeds is not only related to fibre content but also to fibre fermentability and matrix accessibility, which condition microbial utilisation and downstream metabolite production [9, 10]. However, a particular characteristic of nuts and seeds is their compact physical structure. While mastication can extensively reduce the particle size of these food matrices [11], previous in vitro digestion studies showed that further breakdown throughout the gastric and intestinal stages is low, leading to suboptimal digestibility of lipids, e.g. in the range of 8.5–11.3% in almonds [12]. It has also been contrasted that if intestinal conditions are altered, as in the case of CF, matrix degradation, lipolysis, and proteolysis are even lower [11]. Regarding the impact of nuts and seeds consumption on the gut microbiota of children with CF, no information is yet available.

Therefore, the aim of this study was to assess macronutrient digestibility and prebiotic potential of three highly consumed nuts and sunflower seeds through in vitro digestion and colonic fermentation models. In addition, the study aimed to determine possible differences in lipid and protein digestibility and prebiotic potential between healthy and CF gastrointestinal conditions.

## Materials and methods

### Study design

A complete simulation of the digestion of three nuts (peanuts, hazelnuts and almonds) and sunflower seeds was conducted from static in vitro gastrointestinal digestion to static in vitro colonic fermentation. The process was conducted in triplicate (independent digestion tubes per condition) by simulating in parallel for each food the digestion with standard healthy intestinal conditions (H) and under conditions representative of cystic fibrosis (CF) [13]. The composition of macronutrients and dietary fibre according to the label is presented in Table S1. All the foods were raw (not toasted or fried). Aliquots were taken at different timepoints of simulated gastrointestinal digestion (one per tube, i.e., three technical replicates per condition) to determine food matrix degradation, lipolysis and proteolysis. Thereafter, the non-digested fraction was subjected to colonic fermentation with the faecal inoculum of children with cystic fibrosis and microbiota composition and metabolism were analysed. This study design enabled the use of the results from the various analytical determinations as criteria for recommending or discouraging the inclusion of the study foods in the diets of children with cystic fibrosis.

### Materials

Pepsin from porcine gastric mucosa (P6887), pancreatin from porcine pancreas (P7545), bovine bile (B3883), analytical grade salts (potassium chloride, potassium phosphate, sodium bicarbonate, sodium chloride, magnesium chloride hexahydrate, ammonium carbonate and calcium chloride dihydrate), tryptone (T7293), L-cysteine (168,149), sodium sulfide hydrate (14,738), resazurin sodium salt (168,149), sodium phosphate monobasic dihydrate (567,550), dichloromethane (> 99,8%), deuterated chloroform (0.03 vol% TMS), trichloroacetic acid (TCA), tyrosine (≥ 98%, HPLC), ethylenediaminetetraacetic acid (EDTA, 99.4–100.6%), urea (≥ 99.5%), volatile free acid mix (CRM46975), diethylether (99,7%), sulphuric acid (9.2 M), 2-Methylhexanoic acid (52.9 mM), and lactate assay kit (MAK064) were obtained from Sigma-Aldrich (Missouri, USA.). The Enzytec™ Liquid Ammonia (E8390) was provided by R-Biopharm (Darmstadt, Germany). Stool DNA Isolation Kit from Norgen Biotek Corp® (Ontario, Canada) was used. A commercial pancreatic enzyme supplement (Kreon® 10,000 LU, Mylan, Ireland) was provided by Hospital Universitari i Politècnic La Fe, Valencia, Spain. Each capsule of Kreon 10,000 LU contains 150 mg of gastro-resistant microspheres that include porcine pancreatic enzyme equivalent to 10,000 units of lipase, 8000 units of amylase and 600 units of protease. Almonds, hazelnuts, peanuts, and sunflower seeds were purchased at a local supermarket in València (Spain).

### Static in vitro gastrointestinal digestion

In vitro digestion was simulated using two different models: healthy and cystic fibrosis (CF). The healthy model consisted of reproducing the INFOGEST standardised protocol with standard gastrointestinal conditions [14]; and the CF model was based on INFOGEST including modifications to simulate the most critical intestinal alterations found in cystic fibrosis, i.e., reduced pH and bile salts concentration (Table S2) [15], and the use of pancreatic enzyme supplements (Kreon®) instead of the pancreatin reagent used in the healthy model. Simulated digestion fluids, including salivary, gastric and intestinal, were prepared fresh daily from stock solutions [14].

For the oral stage, the simulated salivary fluid and the necessary amount of food containing 2.5 g of fat were added in 1:1 (v/w) ratio to a falcon tube and homogenized with a shaker type of vortex (Heidolph Instruments 541-10,000-00) for 2 min at 37 °C. Foods were previously milled and sieved to simulate mastication ensuring a particle size of less than 2 mm [16]. The gastric stage was simulated by adding the simulated gastric fluid with the oral bolus (1:1 (v/v)), the pH was adjusted with HCl (2N) to pH 3 and tubes were flipped with rotary agitation (Intelli–Mixer RM-2) at 55 rpm for 2 h in an incubation chamber (Boxcult 3,000,957, P Selecta) at 37 °C. Following the gastric stage, simulated intestinal fluid was added to each tube (1:1 (v/v)). Pancreatic enzymes (Kreon® 10,000 lipase units, 8000 amylase units and 600 protease units per capsule) were added to the intestinal stage in the case of the CF condition as a replacement for pancreatin. Depending on the model, the pH was adjusted to pH 7 or 6 using NaOH 1 M. Samples remained in rotatory agitation for 2 h at 37 °C. After the intestinal stage, the temperature of the samples was lowered to inhibit the enzymatic activity and centrifuged (4000 g-force, 20 min at 10 °C). Then, the supernatant was collected and stored at − 20 °C for further analysis, while the solid fraction was mixed with a part of the supernatant as an undigested fraction and kept to simulate the colonic fermentation (explained below).

### Static in vitro colonic fermentation

The simulation was conducted to assess the impact of digested foods (with both S and CF models) on the colonic microbiota composition and metabolism of children with cystic fibrosis. To do so, a pooled faecal inoculum obtained from faecal samples of children with cystic fibrosis was exposed to digested foods. Concretely, static in vitro colonic fermentation was conducted by adding the digested samples to fermentation vials together with culture medium and the faecal inoculum [17]. The experimental design included the simulation of colonic fermentation of all nuts and seeds obtained from both gastrointestinal digestion models; and two types of control samples: i) CF-control: faecal inoculum incubated with digestion fluids corresponding to the CF gastrointestinal model (without food), and (ii) Healthy: faecal inoculum incubated with digestion fluids corresponding to the healthy gastrointestinal model (without food). These controls were included to account for potential effects of the digestion fluids and model-specific conditions (pH, bile salts and enzyme source) on baseline fermentation outcomes.

To prepare the faecal inoculum, paediatric patients with cystic fibrosis (n = 3) were recruited to participate in the project previously accepted by the Ethics Committee of the Universitat Politècnica de València (P03_25-07-2022). The inclusion criteria were age between 3 and 11 years old, a diagnosis of cystic fibrosis confirmed by a positive sweat test (> 60 mEq/L) and/or by the presence of two CF-causing mutations in the CFTR gene, and a confirmed diagnosis of exocrine pancreatic insufficiency (faecal elastase < 200 μg/g in stool). Subjects with antibiotics, prebiotic or probiotic supplements during the last 2 weeks fell within the exclusion criteria. Of note, the use of faecal inocula from only three children may appear restrictive. However, it is important to emphasise that this is not a clinical trial, but an in vitro fermentation study. The use of faecal inocula from three children with CF does not aim to provide representativeness of the CF population, but rather to obtain a microbial ecosystem as ‘starting inocula’, in the same way as enzyme mixes or pH conditions are used to simulate gastrointestinal digestion. In simulated colonic fermentation studies, the faecal donors are not ‘subjects’ but rather ‘biological sources of microbiota’.

To proceed with colonic fermentation, the undigested fraction consisting of 0.5 g of the solid fraction and 10% of the supernatant were added to the sterile fermentation vials, together with 2 mL of faecal inoculum (32% w/v) and 7.5 mL of the culture medium (peptone 15 g/L + 50 ml of reductive solution per 1 L of peptone) and the vials placed in the fermentation chamber (BD Gas Pak™ EZ container) with 2 anaerobic atmosphere generating bags. Samples were incubated for 20 h at 37 °C in a chamber (ICN 120 PLUS, ArgoLab) with an oscillation of 20 rpm using a digital rocking shaker swing (ASW8E, OVAN). After that time, aliquots were taken and stored at − 20 °C until the analytical determinations of microbiota composition and metabolic activity were performed.

### Analytical determinations

#### Matrix degradation index (MDI (%)

MDI (%) is a parameter that estimates the dispersed solids in the digestion tube at the end of the intestinal stage informs about the mechanical disruption on the food matrix along digestion. The total content of a tube was centrifuged at 4000 g-force for 20 min (4 °C) and filtered in a metal sieve (1.6 mm × 1.6 mm mesh) to separate the large particles. The liquid fraction was collected to determine proteolysis, and the solid particles were placed in aluminium dishes in a forced air oven at 60 °C (J.P Selecta 2,000,201) for 48 h until reaching a constant weight. The matrix degradation index was calculated according to [18].

#### Lipolysis extent by nuclear magnetic resonance (NMR)

The lipid fraction of undigested and digested nuts and seeds in the different intestinal conditions was determined by H1 NMR (Nuclear Magnetic Resonance). NMR is a technique that quantifies the lipid species (triglycerides, partial triglycerides and free fatty acids) present in a sample. Undigested nuts and seeds were extracted with dichloromethane in a ratio of 1:2 (w/v) and placed for 1 h in an ultrasonic bath. In contrast, digested samples were extracted with dichloromethane in a ratio of 2:3 (v/v). The tubes were then centrifuged at 6000 g-force for 4 min (20 °C), the content was collected in a volumetric flask, and a saturated solution of NaCl (270 g/L) was added to separate the aqueous fraction from the lipid phase. To recover the lipid fraction and remove the solvent, the samples were introduced into a rotary evaporator. Then, 200 μL of the lipid samples and 400 μL of deuterated chloroform (containing 0.2% non-deuterated chloroform and a small amount [0.03% (v/v)] of tetramethylsilane (TMS)) were mixed in a 5 mm diameter tube. The spectra were recorded on a Bruker Avance 400 spectrophotometer operating at 400 MHz. The spectra acquisition parameters were spectral width 6410 Hz, relaxation delay 3 s, number of scans 64, acquisition time 4.819 s and pulse width 90°. The spectra obtained were analysed with the MestreNova program (V.14; Mnova campus licenses), from the equations validated (Nieva-Echevarría et al. 2014) the molar concentration of triglycerides (TG), diglycerides (1–2 DG and 1–3 DG), monoglycerides (1 MG and 2 MG) and free fatty acids (FA) was determined, the values are provided as average and standard deviation.

#### Proteolysis extent

The extent of proteolysis was determined by characterizing the soluble peptides in trichloroacetic acid (TCA), according to [19]. At the end of digestion, 0.1 mL of the digested samples was added to 0.9 mL of TCA (5% w/v), vortexed, and kept at 4 °C for 1 h. Subsequently, the samples were centrifuged for 10 min at 8000 rpm, and 50 µL of supernatant was added to 1 mL of EDTA-UREA (50 mM EDTA, 8 M urea; pH 10) to measure the absorbance at 280 nm with the DU 730 UV/Vis spectrophotometer from Beckman Coulter (California, USA). A calibration line using tyrosine as standard (0–5 mg/mL) was used to express the results as mg tyrosine equivalents/g of food.

### Microbiota composition by 16S rRNA amplicon gene sequencing

Total DNA was extracted using the Stool DNA Isolation Kit from Norgen Biotek Corp® (Ontario, Canada), and the final yield was determined by Qubit fluorometer. The microbiological analysis was performed by amplifying with specific primers of the V3-V4 regions of the 16S rRNA using Illumina. Amplicons were checked with a Bioanalyzer DNA 1000 chip, and libraries were sequenced using a 2 × 300 bp paired-end run (MiSeq Reagent kit v3) on a MiSeq-Illumina platform (FISABIO sequencing service, Valencia, Spain).

The sequences obtained by sequencing on the Illumina MiSeq platform (2 × 300 bp) were filtered for subsequent analysis. Filtering and quality assessment were performed in FISABIO sequencing service using fastp program [20], on the basis of quality (removal of low-quality nucleotides at the 3′ end, by 10 nucleotides windows with an average quality score under 20) and length (removal of sequences with less than 50 pb). R1 and R2 from Illumina sequences were joined using the FLASH program, applying default parameters. Then, joined sequences were truncated at position 420, and those sequences under that length or having undetermined nucleotides were removed. To analyse the bacterial community by ASVs (Amplicon Sequence Variants), the clean reads were processed in DADA2 package (version 1.32.0) [21]. Exact ASVs were inferred by the DADA2 algorithm, and chimaeras were removed with default parameters. Taxonomy was assigned to ASVs up to the species level, with the SILVA database species train set file (version 138.1) [22]. Phyloseq R package (version 1.48.0) [23] was used to reorganise and manipulate the microbiota data. Microbiota richness and diversity were estimated by calculating the Shannon index for each sample using the microbiome R package (version 1.26.0).

#### Metabolic activity: short-chain fatty acids (SCFA), ammonia and lactate

Short-chain fatty acids (SCFA) were determined according to the protocol of [24]. A 2 mL sample of the colonic fermentation was introduced into a falcon tube, and 0.5 mL of H2SO4 (9.2 M), a small amount of NaCl, 0.4 mL of Internal Standard Solution and 2 mL of diethyl ether were added. The whole mixture was shaken for 1 min, the tube was centrifuged for 3 min at 3000 rpm, and the supernatant was transferred to a chromatography vial for subsequent analysis in a gas chromatograph with flame ionization detector (GC-FID), with a split capillary injector (splitless) and a HP-INNOWAX C30 column (30 m × 0.25 mm × 0.25 μm) (Agilent Technologies, La Jolla, USA). The carrier gas was hydrogen, with a flow rate of 1 ml/min and the detector and injector temperature was 260 °C. The calibration line ranging (0–10 mM) was performed using a mix of volatile fatty acids (Supelco Volatile Free Acid Mix 46,975-U, Sigma-Aldrich). The results were expressed as millimolar concentration (mM), and two groups of SCFA were defined: linear-chain SCFA (lc-SCFA) included the sum of acetic (AA), propionic (PA), butyric (BA), caproic (CA), and valeric acid (VA); branched-chain SCFA (bc-SCFA) considered as the sum of isovaleric acid (IVA), isocaproic acid (ICA), and isobutyric acid (IBA).

For the determination of the ammonia concentration, the commercial enzymatic kit Enzytec™ Liquid Ammonia from R-Biopharm (Darmstadt, Germany) was used. The samples were previously deproteinized with the commercial enzymatic kit Deproteinizing Sample Preparation Kit from Sigma-Aldrich (Missouri, USA) following the manufacturer’s instructions. The results were expressed as millimolar concentration (mM). The commercial enzymatic kit Lactate Assay from Sigma-Aldrich (Missouri, USA) was used to determine lactate concentration. The results were expressed as micromolar concentration (μM).

### Statistical analyses

Data were summarised as mean and standard deviation obtained from three replicates to present the descriptive results: MDI, proteolysis, lipolysis, lipid species, microbiota composition at phylum and genus taxonomic levels (relative abundance) and concentration of metabolites (SCFA, lactate and ammonia). One-way ANOVAs were applied to compare MDI, proteolysis and lipolysis in the nuts and seed samples digested under CF or healthy conditions (Statgraphics Centurion XVIII Software® (Virginia, USA) version 19.6.03 (one-way ANOVA) (*p*-value > 0.05)). Differences in microbiota composition and metabolic activity between digestion conditions (healthy vs. CF) and food samples (almond, hazelnut, peanut and sunflower seed), were assessed in terms of beta-diversity (ordination model PCoA, distance method Bray–Curtis Index, and statistical method PERMANOVA) using Microbiome Analyst online tool (https://www.microbiomeanalyst.ca, last accessed 22-01-2026). Additionally, differential abundance analysis at phylum and genus levels was performed using the ANCOM-BC method implemented in MicrobiomeAnalyst (https://www.microbiomeanalyst.ca). Analyses were conducted separately for each digestion model (healthy and CF), with each food matrix compared against its corresponding control. This approach accounted for the compositional nature of microbiome data and provided robust estimates of differential abundance. Statistical significance was determined using false discovery rate (FDR)-adjusted *p*-values (q < 0.05) based on the Benjamini–Hochberg correction.

## Results and discussion

### Digestibility after simulated gastrointestinal digestion

**Fig. 1:**
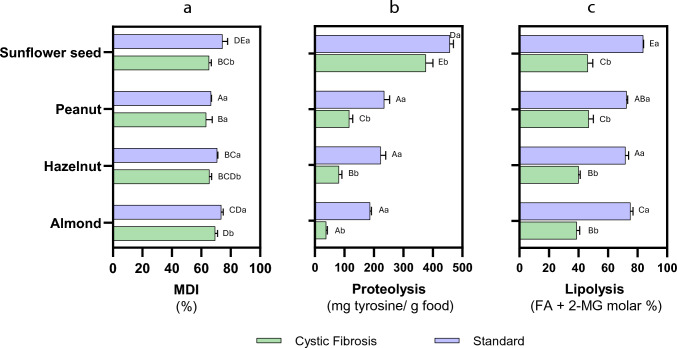
Matrix degradation index (MDI (%)) **a**, proteolysis (mg tyrosine/g food) **b** and lipolysis (%) **c** after in vitro simulated gastrointestinal digestion of nuts and seeds (almond, hazelnut, peanut and sunflower seed) under different intestinal conditions (healthy and cystic fibrosis). A–E: Different capital letters indicate significant differences between the different foods under each gastrointestinal condition at a significance level of 95% (*p* < 0.05). **a**–**b**: Different lowercase letters indicate significant differences between the two digestion models for the same food at a confidence level of 95% (*p* < 0.05). Data met the assumptions of normality and homogeneity of variances (Shapiro–Wilk and Levene’s tests)

The digestibility of the different samples was indirectly assessed by quantifying the matrix degradation index (MDI), and proteolysis and lipolysis extent. Focusing first on the MDI (Fig. 1a), all samples reached values between 67 and 75% when digested under healthy simulated digestion conditions, these values being slightly lower, but statistically significant, with exception of peanut, under conditions of CF (< 69%). The MDI provides information on the mechanical breakdown of the food matrix during digestion [11] as it is dierctly related with the surface area of nutrients exposed to digestive fluids. However, even with the optimal conditions of the healthy model, lipolysis and proteolysis extents did not reach 100%, according to previous literature [25].

Concerning protein digestibility (soluble protein TCA) (Fig. 1b), the highest values (187–457 mg tyrosine/ g food) were found under the healthy GI conditions, as expected. If CF and S digestive conditions are compared in terms of proteolysis extent, sunflower seeds showed the highest protein hydrolysis (457 vs. 376 mg tyrosine/g food) and the lowest relative variation (− 18%) between both models. The protein bodies of sunflower seeds are visibly noticeable as round globules and are densely distributed in the cells [26]. This cell structure facilitates the digestibility of the protein, which has a good balance in terms of amino acid composition, such as alanine, glycine, glutamic acid, leucine and aspartic acid [27]. In peanut, the extent of proteolysis was not affected by pH and bile concentration, in contrast to the other samples. In contrast, almond protein was the least digestible regardless of the gastrointestinal conditions and the most affected by the CF as (187 vs. 38 mg tyrosine/g food for S and CF model, respectively). The higher presence of anti-nutrients such as phytates in almonds (350 mg/100 g) [28] could be responsible for the reduced proteolysis found, as phytates have shown evidence of forming complexes with proteins, resulting in decreased proteolytic enzyme activity and digestibility [29].

Moving onto lipolysis, considered as the sum of the products free fatty acids (FA) and 2-monoglycerols (2-MG), significantly different values were also observed when comparing both digestion models (S vs. CF) (Fig. 1c). In the CF model, sunflower seed and peanut reached the highest values, approximately 46% of lipolysis extent, and hazelnut and almond were in the range of 40% and 39%, respectively. In contrast, the S model enabled significantly higher values in the range of 72 and 84%. This expected result is explained by the fact that lipase activity decreases at pH 6 (compared to optimal pH 7); besides the low bile salts concentration (1 mM instead of 10 mM) prevents from efficient lipid micellization, resulting in reduced surface area of exposure of lipid droplets to pancreatic enzymes [30]. Reduced lipolysis after simulated intestinal digestion with unfavourable conditions of cystic fibrosis has been repeatedly confirmed for other lipid and protein-rich food matrices [4, 13].

**Fig. 2:**
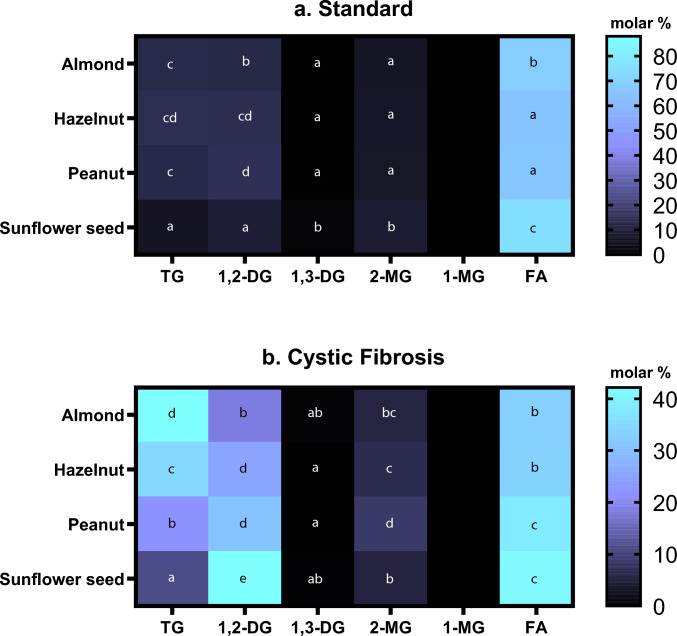
Lipid species distribution (molar concentration (%)) after in vitro simulated gastrointestinal digestion of nuts and seeds (almond, hazelnut, peanut and sunflower seed) at different intestinal conditions (Healthy (a) and Cystic Fibrosis (b)). TG: triglycerides; 1,2-DG: 1–2 diglycerides; 1,3-DG: 1–3 diglycerides; 2-MG: 2-monoglycerides; 1-MG: monoglycerides; FA: fatty acids. a-g: Different lowercase letters represent significant differences between nuts and seed in each lipid fraction at 95% confidence level (*p* < 0.05). Data met the assumptions of normality and homogeneity of variances (Shapiro–Wilk and Levene’s tests)

Complementary to the quantification of total lipolysis extent, the complete lipid species profile was established (Fig. 2a). At the healthy digestion model, the results showed that most lipid species in all the samples were free fatty acids (65–75%), i.e., the final product of triglyceride digestion; while triglycerides, diglycerides and monoglycerides were represented in low proportions. The food matrix showing the significantly higher FA proportions was sunflower seed (75.50%), while hazelnut had the lowest (65.11%). Nuts and seeds after simulated digestion with the CF model (Fig. 2b) showed differences between matrices. Sunflower seeds had the lowest proportion of triglycerides but comparable FA (41%) to almond, hazelnut, or peanut, at the expense of increased values of 1,2-DG. Sunflower seeds and peanuts seemed to be more easily digested [26].

Therefore, despite the challenges of fat maldigestion associated with CF, sunflower seeds and peanuts could be a valuable food for contributing to a high-calorie and high-fat diet that supports overall health and weight maintenance, along with high protein and dietary fibre [3].

### Change in microbiota diversity and composition after simulated colonic fermentation

The impact of the undigested fraction of the different nuts and seeds obtained under CF and healthy luminal conditions on CF donor microbiota was assessed. The microbiota composition of the control in both CF and healthy conditions was composed mainly by Bacteroidota (former Bacteroidetes), Bacillota (former Firmicutes) and Pseudomonadota (former Proteobacteria). At the genus taxonomic level, the composition was dominated by *Bacteroides*, *Acidaminococcus*, *Alistipes*, *Sutterella*, *Parabacteroides*, *Prevotella* and *Subdoligranulum*. In terms of diversity, the healthy control was characterised by alpha diversity with Shannon Index of 5.10 (0.01) and Chao-1 Index of 730.50 (13.50); while CF control presented alpha diversity with Shannon Index of 5.15 (0.01) and Chao-1 Index of 718.50 (20.50) (Table S3).

**Fig. 3:**
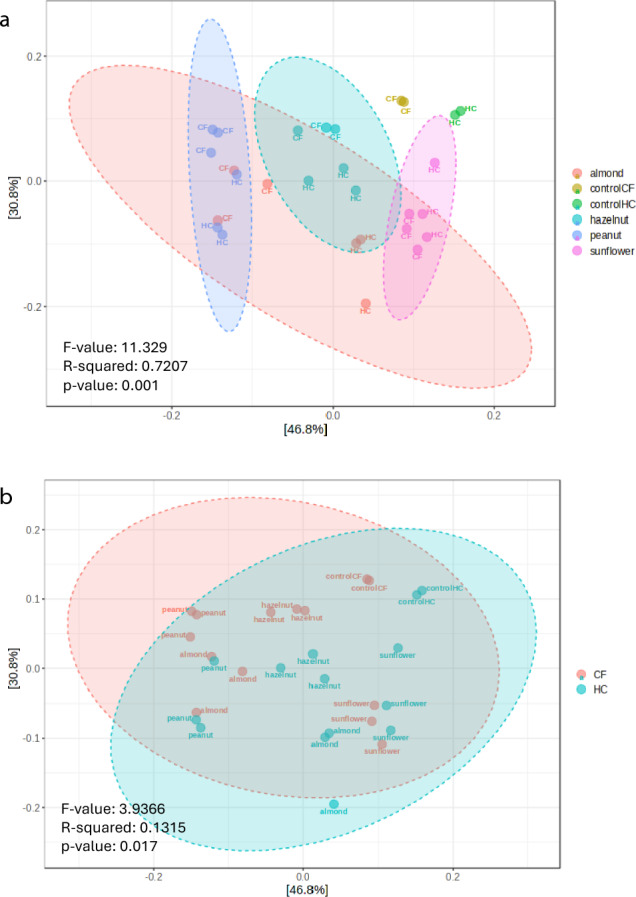
Microbiota diversity after simulated colonic fermentation: **a** PCoA plot of beta diversity showing sample grouping by food (almond, peanut, hazelnut, sunflower seed); **b** PCoA plot of beta diversity showing sample grouping by simulated condition (CF, cystic fibrosis; HC, healthy control). Statistical parameters are obtained from distance method Bray–Curtis Index, and statistical method PERMANOVA

After the exposure of the basal microbiota during simulated colonic fermentation to the undigested fraction of nuts and sunflower seeds, significant differences were observed in terms of beta-diversity. The analysis revealed different patterns in microbiota composition resulted after colonic fermentation between the four food samples (F-value: 3.9366; R-squared: 0.1315; FDR *p*-value: 0.017) (Fig. 3a) and also considering the two simulated gastrointestinal conditions (healthy vs. CF) (F-value: 11.329; R-squared: 0.72027; FDR *p*-value: 0.001) (Fig. 3b).

**Fig. 4:**
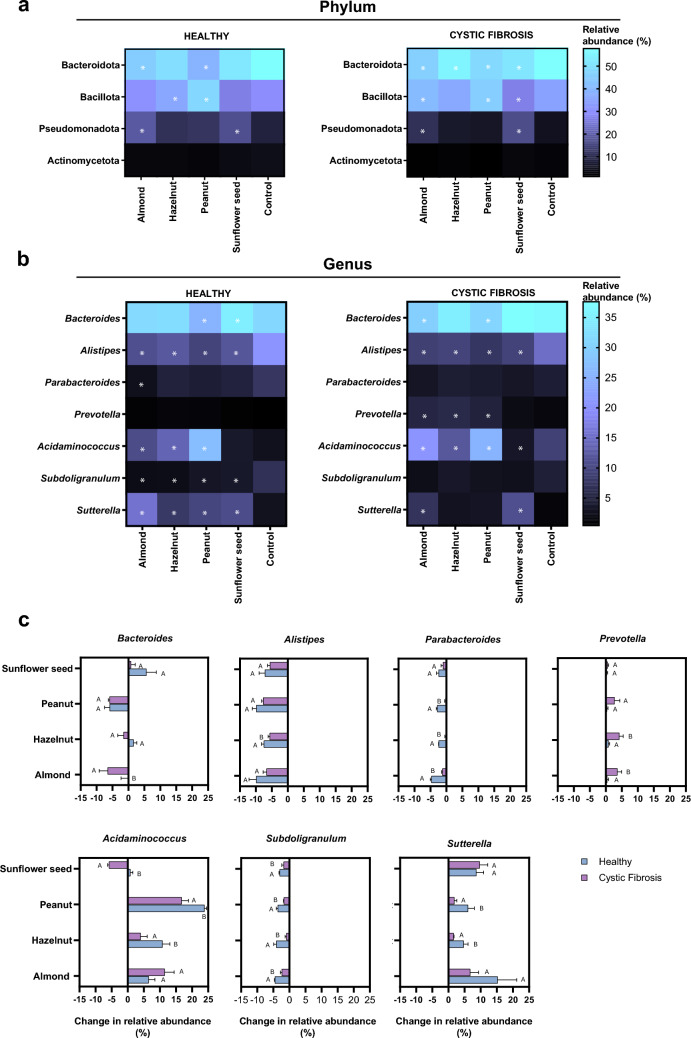
Relative abundance of microbiota composition at phylum **a** and genus **b**, **c** level after in vitro colonic fermentation of nuts and seeds under different intestinal conditions (Healthy and Cystic Fibrosis). (*) Statistically significant differences compared to the corresponding control within each digestion model, identified using differential abundance analysis (ANCOM-BC, q < 0.05). The changes in relative abundance (%) of different taxa are shown for both conditions

In addition, different effects in composition at the phylum level and different patterns depending on the gastrointestinal digestion model were noted (Table S4) (Fig. 4a). In general, in both models, nuts and the seed led to decreased Bacteroidota (more decreased in healthy conditions) and increased Bacillota and Pseudomonadota. Sunflower seed stood out in the CF conditions for being the only matrix inducing a significant decrease in Bacillota (− 7.5%), and for imparting the highest increase in Pseudomonota (+ 11.9%).

Moving onto changes at genus level that the digested nuts and seed imparted to colonic microbiota composition (Table S5) (Fig. 4b, c**)**, the most relevant observations were that sunflower seeds were effective in reducing *Acidaminococcus* in the CF model (− 5.83%) and supported *Bacteroides* increase in the healthy model (+ 5.65%). This is considered a positive result, as the genus *Acidaminococcus* has been linked to intestinal inflammation and lower growth rates in children [31], while *Bacteroides* is a major producer of short-chain fatty acids that exert a beneficial effect on modulating intestinal inflammation [32]. The relative abundance of *Alistipes* decreased with all nuts and sunflower seeds in both conditions by approximately 5–10%, and *Sutterella* significantly increased with all foods in the healthy model and with almond and sunflower seeds in CF conditions. These could be considered as negative results, since *Alistipes* species are producers of dietary succinate, promoting glucose homeostatic balance [33]; and *Sutterella* is a pro-inflammatory opportunistic bacterium [34].

A limitation of the batch fermentation approach to be acknowledged is the simplified culture medium employed (peptone-based), which does not fully reproduce the complexity of the colonic ecosystem, including host-derived substrates such as mucins and the baseline presence of fermentation end-products. Such factors can modulate the competitive fitness of specific bacterial groups and may influence community-level shifts observed in vitro. Therefore, our findings should be interpreted as a controlled mechanistic screening, and confirmation in dietary interventions or well-characterised in vivo studies is warranted.

### Short-chain fatty acids, lactate and ammonia production after simulated colonic fermentation

**Fig. 5:**
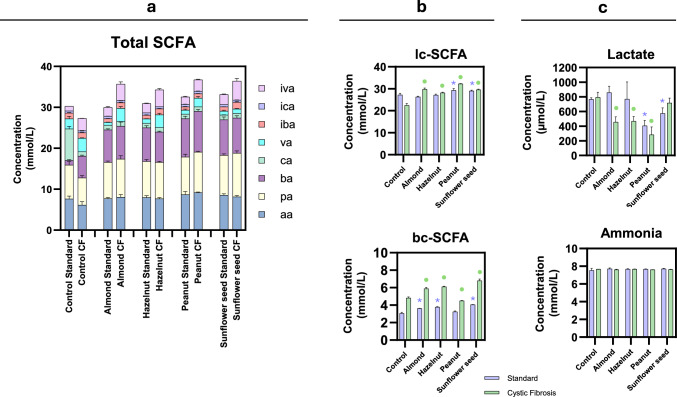
Metabolite production during static in vitro simulation of colonic fermentation: **a** total short chain fatty acids (aa: acetic acid, pa: propionic acid, ba: butyric acid, ca: caproic acid, va: valeric acid, iba: isobutyric acid, ica: isocaproic acid and iva: isovaleric acid), Total SCFA; **b** sum of linear chain fatty acids, lc-SCFA, and sum of branched chain fatty acids, bc-SCFA; **c** lactate and ammonia. (*) Statistically significant differences (ANOVA) of all nuts and seed compared to the control of healthy model at a confidence level of 95% (*p* < 0.05). (●) Statistically significant differences (ANOVA) of all nuts and seed compared to the control of cystic fibrosis model at a confidence level of 95% (*p* < 0.05). Data met the assumptions of normality and homogeneity of variances (Shapiro–Wilk and Levene’s tests)

The production of SCFA because of colonic fermentation of nuts and seeds was found in a concentration range from 30 to 35 mM, with a general observation of higher values in CF than in healthy conditions, even though in the control situation, the concentration was lower in CF (Fig. 5a). After colonic fermentation, most of the nuts and seed led to a similar abundance of specific SCFA in both conditions, in the sense that butyric, propionic and acetic were the most abundant. The formation of lc-SCFA could provide health and nutritional benefits for the host [35], and these were significantly increased in both CF and healthy conditions. However, focusing on the series of the bc-SCFA (especially isovaleric acid) higher values were found in most of nuts and seed in the CF conditions, this difference explaining the overall higher total SCFA concentration (Fig. 5b). The bc-SCFA are produced by the fermentation of branched amino acids, and are therefore commonly considered markers of proteolytic fermentation [36]. Thus, in the present CF digestion model, the higher amount of undigested protein potentially reaching the colonic compartment (due to pancreatic insufficiency) could partially explain the increased bc-SCFA levels [37]. The biological effects of bc-SCFA in CF and non-CF populations is not entirely clear in the literature and more studies are needed [35, 37]. However, in other contexts, the production of bc-SCFA has been associated with harmful effects related to poor gut health, including, an increased contribution of proteolytic fermentation, higher generation of potentially toxic metabolites such as indoles, and associations with intestinal inflammation and epithelial dysfunction. Elevated bc-SCFA levels are therefore often interpreted as markers of a shift towards protein-driven fermentation, which is generally considered less favourable than carbohydrate-driven saccharolytic fermentation [38].

The ammonia production (Fig. 5c) was evenly distributed in all nuts and seeds and intestinal digestion models, with no statistically significant differences with respect to control values (8 mM). However, in the case of lactate, CF intestinal conditions led to decreased concentration compared to the control, except with the sunflower seed, in which lactate increased. A similar tendency towards reduced lactate production was observed in the series of healthy model experiments. This could be due to the presence of intestinal microorganisms that can utilise lactate to convert it into short-chain fatty acids [31]. According to our results, the production of lc-SCFA (acetate, butyrate and propionate) was significantly increased. This was also evident in the context of healthy conditions, where lactate concentration was inversely proportional to lc-SCFA concentration.

## Conclusion

This in vitro study provides a valuable screening approach to inform potential dietary recommendations for children with cystic fibrosis (CF). Among the evaluated foods, sunflower seeds and peanuts exhibited the highest degree of lipolysis, with sunflower seeds also achieving the greatest extent of proteolysis. Notably, sunflower seeds had a distinct effect on the colonic microbiota, being the only food associated with a decrease in *Acidaminococcus* and the smallest increase in *Prevotella* abundance. Despite promoting the production of branched-chain short-chain fatty acids (bc-SCFAs)—as observed with the other nuts and seeds—sunflower seeds also led to increased levels of linear-chain SCFAs (lc-SCFAs) and lactate. However, the directionality of some shifts is not unequivocally beneficial in the CF context. Thus, sunflower seeds may represent a candidate food matrix for further investigation in well-designed dietary intervention studies rather than a direct dietary recommendation based solely on in vitro data.

## Supplementary Information

Below is the link to the electronic supplementary material.

Supplementary file1 (DOCX 31 KB)

## Data Availability

Data will be made available upon request.
