# Supplementary material for: Digestibility and microbiota modulation by nuts and sunflower seeds in cystic fibrosis: an in vitro approach
Source: Eur J Nutr. 2026 Jun 6;65(4):154. doi: 10.1007/s00394-026-04006-7 (PMC13242477; doi:10.1007/s00394-026-04006-7)
Supplement: Supplementary file 1 — Supplementary file1 (DOCX 31 KB) [file 394_2026_4006_MOESM1_ESM.docx]

**Table S1.** Macronutrient composition of nuts and seeds in terms of fat, carbohydrates, protein and fibre contents expressed in g/100g of food.

| **Food** | **Fat** | **Carbohydrates** | **Protein** | **Fibre** |
| --- | --- | --- | --- | --- |
| Almond | 50.00 | 21.0 | 21.0 | 12.0 |
| Hazelnut | 60.0 | 18.0 | 15.0 | 9.0 |
| Peanut | 50.0 | 6.2 | 29.0 | 11.0 |
| Sunflower seed | 50.0 | 18.0 | 22.0 | 18.0 |

**Table S2.** Established parameters in the gastrointestinal stage for the standard and cystic fibrosis models.

| **Digestion model** |  | **Oral** | **Gastric** | **Intestinal** |  |
| --- | --- | --- | --- | --- | --- |
| Standard (S) | Digestion fluid | Simulated  salivary fluid (SSF) | Simulated  gastric  fluid (SGF) | Simulated  intestinal  fluid (SIF) |  |
|  | Digestion conditions | 5 min pH 7 | 2 h pH 3 | 2 h pH 7 |  |
|  | Enzymatic concentration | - | Pepsin:  20000 U/mL | Pancreatin:  2000 U/mL |  |
|  | Bile salts concentration | - | - | 10 mM in the  intestinal  volume (77.18  mg of bovine extract / mL) |  |
| Cystic fibrosis (CF) | Digestion fluid | SSF | SGF | SIF |  |
|  | | Digestion conditions | 5 min pH 7 | 2 h pH 3 | 2 h pH 6 |
|  | | Enzymatic concentration | - | Pepsin:  20000 U/mL | Pancreatin (Kreon®):  2000 LU/g of fat |
|  | | Bile salts concentration | - | - | 1 mM in the  intestinal  volume (7.72  mg of bovine extract / mL) |

**Table S3.** α diversity (Shannon Index and Chao-1 Index) of the faecal microbiota after *in vitro* colonic fermentation of the different nuts and seeds under each digestion model (S and CF). Asterisk (*) shows the significant differences with respect to the control in each digestion model.

| **Digestion model** | **Nuts and seeds** | **Shannon Index**  **(mean (SD))** | **Chao-1 Index**  **(mean (SD))** |
| --- | --- | --- | --- |
| Standard | Control | 5.10 (0.01) | 730.50 (13.50) |
|  | Almond | 4.97 (0.06) | 876.67 (65.50) |
|  | Hazelnut | 5.13 (0.05) | 899.33 (102.18) ^*^ |
|  | Peanut | 4.88 (0.12) ^***^ | 825.00 (102.43) |
|  | Sunflower seed | 5.12 (0.01) | 777.33 (59.35) |
| Cystic fibrosis | Control | 5.15 (0.01) | 718.50 (20.50) |
|  | Almond | 5.02 (0.01) | 916.00 (109.53) ^**^ |
|  | Hazelnut | 5.22 (0.01) | 896.00 (86.71) ^*^ |
|  | Peanut | 4.93 (0.07) ^***^ | 815.67 (88.95) |
|  | Sunflower seed | 5.14 (0.05) | 815.00 (42.57) |

**Table S4.** Statistically significant differences (ANCOM-BC) in faecal microbiota bacterial phylum after *in vitro* colonic fermentation of the different nuts and seeds under each digestion model (S and CF) compared to the control.

| **Digestion model** | **Taxon** | **Comparison** | **Control (%)** | **Treatment (%)** | **Direction** | **q-value** |
| --- | --- | --- | --- | --- | --- | --- |
| CF | Bacteroidota | Almond vs control | 57.75 | 46.85 | Decreased | 0.00013 |
| CF | Bacteroidota | Peanut vs control | 57.75 | 47.11 | Decreased | 0.00013 |
| CF | Bacteroidota | Sunflower seed vs control | 57.75 | 52.81 | Decreased | 0.00022 |
| CF | Bacillota | Almond vs control | 34.51 | 39.43 | Increased | 0.00022 |
| CF | Bacillota | Peanut vs control | 34.51 | 45.43 | Increased | 0.00013 |
| CF | Bacillota | Sunflower seed vs control | 34.51 | 27.01 | Decreased | 0.00013 |
| CF | Pseudomonadota | Almond vs control | 4.59 | 11.57 | Increased | 0.00013 |
| CF | Pseudomonadota | Sunflower seed vs control | 4.59 | 16.48 | Increased | 0.00013 |
| Healthy | Bacteroidota | Almond vs control | 57.09 | 42.80 | Decreased | 0.00013 |
| Healthy | Bacteroidota | Peanut vs control | 57.09 | 38.96 | Decreased | 0.00013 |
| Healthy | Bacillota | Hazelnut vs control | 29.50 | 34.83 | Increased | 0.02000 |
| Healthy | Bacillota | Peanut vs control | 29.50 | 46.29 | Increased | 0.00013 |
| Healthy | Pseudomonadota | Almond vs control | 8.05 | 22.08 | Increased | 0.00013 |
| Healthy | Pseudomonadota | Sunflower seed vs control | 8.05 | 15.04 | Increased | 0.00013 |

**Table S5.** Statistically significant differences (ANCOM-BC) in faecal microbiota bacterial genus after *in vitro* colonic fermentation of the different nuts and seeds under each digestion model (S and CF) compared to the control.

| **Digestion model** | **Taxon** | **Comparison** | **Control (%)** | **Treatment (%)** | **Direction** | **q-value** |
| --- | --- | --- | --- | --- | --- | --- |
| Healthy | *Bacteroides* | Peanut vs control | 30.46 | 24.57 | Decreased | 0.00019 |
| Healthy | *Bacteroides* | Sunflower seed vs control | 30.46 | 36.11 | Increased | 0.00019 |
| Healthy | *Acidaminococcus* | Almond vs control | 2.39 | 8.90 | Increased | 0.00019 |
| Healthy | *Acidaminococcus* | Hazelnut vs control | 2.39 | 13.22 | Increased | 0.00019 |
| Healthy | *Acidaminococcus* | Peanut vs control | 2.39 | 26.24 | Increased | 0.00019 |
| Healthy | *Alistipes* | Almond vs control | 18.75 | 8.86 | Decreased | 0.00019 |
| Healthy | *Alistipes* | Hazelnut vs control | 18.75 | 11.26 | Decreased | 0.00019 |
| Healthy | *Alistipes* | Peanut vs control | 18.75 | 8.89 | Decreased | 0.00019 |
| Healthy | *Alistipes* | Sunflower seed vs control | 18.75 | 11.61 | Decreased | 0.00019 |
| Healthy | *Sutterella* | Almond vs control | 2.50 | 17.75 | Increased | 0.00019 |
| Healthy | *Sutterella* | Hazelnut vs control | 2.50 | 7.22 | Increased | 0.00026 |
| Healthy | *Sutterella* | Peanut vs control | 2.50 | 7.92 | Increased | 0.00019 |
| Healthy | *Sutterella* | Sunflower seed vs control | 2.50 | 9.16 | Increased | 0.00019 |
| Healthy | *Parabacteroides* | Almond vs control | 6.92 | 2.23 | Decreased | 0.00038 |
| Healthy | *Subdoligranulum* | Almond vs control | 6.33 | 1.83 | Decreased | 0.00061 |
| Healthy | *Subdoligranulum* | Hazelnut vs control | 6.33 | 2.26 | Decreased | 0.00246 |
| Healthy | *Subdoligranulum* | Peanut vs control | 6.33 | 2.73 | Decreased | 0.00943 |
| Healthy | *Subdoligranulum* | Sunflower seed vs control | 6.33 | 3.32 | Decreased | 0.04200 |
| CF | *Bacteroides* | Almond vs control | 36.50 | 29.97 | Decreased | 0.00019 |
| CF | *Bacteroides* | Peanut vs control | 36.50 | 30.59 | Decreased | 0.01721 |
| CF | *Acidaminococcus* | Almond vs control | 9.06 | 20.54 | Increased | 0.00019 |
| CF | *Acidaminococcus* | Hazelnut vs control | 9.06 | 13.06 | Increased | 0.00019 |
| CF | *Acidaminococcus* | Peanut vs control | 9.06 | 25.82 | Increased | 0.00019 |
| CF | *Acidaminococcus* | Sunflower seed vs control | 9.06 | 3.23 | Decreased | 0.00019 |
| CF | *Alistipes* | Almond vs control | 15.17 | 8.50 | Decreased | 0.00019 |
| CF | *Alistipes* | Hazelnut vs control | 15.17 | 9.51 | Decreased | 0.00019 |
| CF | *Alistipes* | Peanut vs control | 15.17 | 7.45 | Decreased | 0.00019 |
| CF | *Alistipes* | Sunflower seed vs control | 15.17 | 9.57 | Decreased | 0.00019 |
| CF | *Sutterella* | Almond vs control | 1.17 | 7.93 | Increased | 0.00019 |
| CF | *Sutterella* | Sunflower seed vs control | 1.17 | 10.86 | Increased | 0.00019 |
| CF | *Prevotella* | Almond vs control | 1.34 | 4.97 | Increased | 0.00019 |
| CF | *Prevotella* | Hazelnut vs control | 1.34 | 5.57 | Increased | 0.00019 |
| CF | *Prevotella* | Peanut vs control | 1.34 | 4.05 | Increased | 0.00431 |
| CF | *Subdoligranulum* | Almond vs control | 4.47 | 2.12 | Decreased | 0.01721 |

**Figure S1.** Microbiota composition of the control in terms of phylum level; D1: Donor #1, D2: Donor #2, D3: Donor #3, I: Inoculum.
